# The promise and limitations of artificial intelligence in CTPA-based pulmonary embolism detection

**DOI:** 10.3389/fmed.2025.1514931

**Published:** 2025-03-19

**Authors:** Lin Li, Min Peng, Yifang Zou, Yunxin Li, Peng Qiao

**Affiliations:** ^1^Department of Radiology, Yantaishan Hospital, Yantai, China; ^2^Department of Radiology, Yantai Yuhuangding Hospital, Affiliated Hospital of Qingdao University, Yantai, China; ^3^Department of Equipment, Yantaishan Hospital, Yantai, China

**Keywords:** pulmonary embolism, artificial intelligence, diagnostic imaging, machine learning, clinical applications

## Abstract

Computed tomography pulmonary angiography (CTPA) is an essential diagnostic tool for identifying pulmonary embolism (PE). The integration of AI has significantly advanced CTPA-based PE detection, enhancing diagnostic accuracy and efficiency. This review investigates the growing role of AI in the diagnosis of pulmonary embolism using CTPA imaging. The review examines the capabilities of AI algorithms, particularly deep learning models, in analyzing CTPA images for PE detection. It assesses their sensitivity and specificity compared to human radiologists. AI systems, using large datasets and complex neural networks, demonstrate remarkable proficiency in identifying subtle signs of PE, aiding clinicians in timely and accurate diagnosis. In addition, AI-powered CTPA analysis shows promise in risk stratification, prognosis prediction, and treatment optimization for PE patients. Automated image interpretation and quantitative analysis facilitate rapid triage of suspected cases, enabling prompt intervention and reducing diagnostic delays. Despite these advancements, several limitations remain, including algorithm bias, interpretability issues, and the necessity for rigorous validation, which hinder widespread adoption in clinical practice. Furthermore, integrating AI into existing healthcare systems requires careful consideration of regulatory, ethical, and legal implications. In conclusion, AI-driven CTPA-based PE detection presents unprecedented opportunities to enhance diagnostic precision and efficiency. However, addressing the associated limitations is critical for safe and effective implementation in routine clinical practice. Successful utilization of AI in revolutionizing PE care necessitates close collaboration among researchers, medical professionals, and regulatory organizations.

## 1 Introduction

Pulmonary embolism is a potentially fatal illness that occurs when a blood clot (embolus) travels through the bloodstream and lodges in the lungs’ arteries. This obstruction can impede blood flow to the lungs, leading to serious complications such as respiratory failure, pulmonary infarction, or even death if not promptly diagnosed and treated. Pulmonary embolism (PE) frequently occurs due to deep vein thrombosis (DVT), which involves the formation of blood clots in the deep veins of the legs or pelvis. These clots subsequently break and move to the lungs (1). In clinical practice, PE presents a significant challenge due to its nonspecific symptoms, which can mimic other conditions such as heart attack or pneumonia. Common symptoms include shortness of breath, sudden chest pain, rapid heartbeat, and coughing blood. However, not all patients experience typical symptoms, making diagnosis even more challenging (2). As a result, healthcare providers rely on clinical assessment, laboratory tests (such as D-dimer), and imaging studies to confirm the diagnosis. Given the potential severity of PE and the need for rapid intervention, timely diagnosis is critical (3). The standard course of treatment usually consists of administering anticoagulant medication to prevent the formation of more blood clots and facilitate the body’s natural process of dissolving existing clots. In severe cases, more intrusive measures may be required, such as thrombolytic treatment, surgical embolectomy, catheter directed thrombolysis, thrombus aspiration, mechanical thrombectomy, and extracorporeal membrane oxygenation (4–6). Therefore, in medical practice, accurately identifying the indications and manifestations of PE and timely diagnosis and suitable treatment may substantially improve patient outcomes and decrease the probability of complications (7).

Computed tomography pulmonary angiography (CTPA) is an advanced imaging method employed for diagnosing PE by visually representing the blood arteries in the lungs. It involves the injection of a contrast dye into a vein, followed by rapid imaging using a computed tomography scanner. The contrast dye highlights the blood vessels, allowing radiologists to identify any blockages caused by blood clots (8). It can accurately detect the location and extent of blood clots within the pulmonary arteries, assisting medical professionals in making prompt and well-informed treatment decisions (9). Moreover, CTPA is non-invasive and relatively quick, making it a preferred imaging method in emergencies where prompt diagnosis is essential. Despite its advantages, CTPA does have some limitations, including its reliance on ionizing radiation and the use of contrast dye, which may pose risks for some patients, such as those with kidney disease or allergies (10). Additionally, overuse of CTPA can lead to unnecessary radiation exposure and healthcare costs. For these reasons, healthcare providers must weigh the benefits and risks of CTPA on a case-by-case basis and consider alternative imaging methods when appropriate.

Artificial intelligence (AI) is transforming the domain of medical imaging and diagnostics, providing novel prospects to enhance precision, efficacy, and patient welfare. Over the past few years, significant progress has been made in developing and training AI algorithms to efficiently and accurately assess medical images, including MRIs, CT scans, and X-rays (11, 12). AI systems can recognize abnormalities, find patterns, and aid radiologists in making more precise diagnoses (13). In the context of PE, AI can enhance the interpretation of imaging studies, such as CTPA, by assisting radiologists in identifying subtle signs of PE that may be overlooked or misinterpreted (14). AI systems can more quickly examine vast amounts of imaging data than human radiologists. This allows for expedited diagnosis and the prompt beginning of treatment. Also, AI can integrate data from multiple sources, including imaging studies, patient medical records, and laboratory tests, to provide a comprehensive picture of the patient’s health status. Table 1 presents the main AI methods used in CTPA-based PE detection.

**Table 1:** Overview of different AI techniques used in CTPA-based pulmonary embolism detection.

AI Technique	Description	Advantages	Limitations	Reference
Deep learning	Uses deep neural networks for feature extraction and classification	High accuracy, adaptable to data	Requires large datasets, computationally expensive	(13)
Convolutional neural networks (CNNs)	Specialized deep learning models for image analysis	Effective in image recognition tasks	Need for labeled training data	(30)
Support vector machines (SVM)	Supervised learning algorithm for classification and regression	Effective with high-dimensional data	Limited scalability to large datasets	(77)
Random forest	Ensemble learning approach combining multiple decision trees	Robust to overfitting, handles non-linear data	May overfit with noisy data	(78)
Gradient boosting machines	Sequentially adds weak learners to minimize loss	High predictive accuracy	Sensitive to noisy data	(79)
Genetic algorithms	Optimization techniques inspired by natural selection	Effective in global optimization	Computationally intensive	(80)

By leveraging this wealth of information, AI systems can assist healthcare providers in making personalized treatment recommendations tailored to each patient’s unique needs and characteristics (15, 16). Despite its potential advantages, the integration of AI into clinical practice presents several challenges. These include the necessity for comprehensive validation studies to establish the reliability and safety of AI algorithms, as well as concerns related to data privacy, regulatory approval, and reimbursement. It is imperative for healthcare providers to adopt AI technologies while ensuring their effective incorporation into existing clinical workflows.

This review article aims to comprehensively examine the current landscape of CTPA-based pulmonary embolism detection, focusing specifically on the thriving role of AI in this domain. Through a critical analysis, we elucidate the capabilities and limitations of AI-driven approaches in detecting pulmonary embolisms from CTPA scans, exploring key advancements, challenges, and potential future directions. This review will provide valuable insights for clinicians, researchers, and technologists involved in developing and implementing AI technologies in pulmonary embolism diagnosis by summarizing the existing studies and knowledge in the field.

## 2 CTPA in PE detection

### 2.1 Principles and procedure of CTPA

A non-invasive imaging method called CTPA is mostly used to diagnose PE, a potentially fatal condition caused by blockage of one or more pulmonary arteries in the lungs. The procedure involves using a CT scanner to generate detailed cross-sectional images of the pulmonary arteries. The principle behind CTPA lies in the intravenous injection of contrast material, typically iodine-based, which highlights the blood vessels within the lungs, allowing for clear visualization of any potential blockages or abnormalities (17). The CT scanner then captures images as the contrast material flows through the pulmonary vasculature. To perform a CTPA, the patient is positioned on the CT scanner table, usually lying flat on their back. An intravenous line is inserted, typically in the arm, through which the contrast material is injected (18). While quickly taking X-ray photographs, the scanner table travels through the CT machine. Usually, the entire process takes a few minutes. Once the images are obtained, specialized software is used to reconstruct them into detailed 3D images that can be analyzed by a radiologist for the presence of pulmonary embolism or other lung conditions.

### 2.2 Advantages and challenges of CTPA in diagnosing PE

CTPA offers several advantages in diagnosing pulmonary embolism compared to other imaging modalities (19). Firstly, it provides high-resolution images that allow for accurate visualization of even small emboli within the pulmonary arteries. This enables prompt and precise diagnosis, which is of primary relevance in managing PE, associated with significant morbidity and mortality (20). Additionally, CTPA is a non-invasive procedure, which means it does not require the insertion of catheters into the blood vessels, unlike conventional pulmonary angiography. This lowers the possibility of issues and makes it a safer choice, especially for individuals who should not have intrusive operations done.

Nevertheless, CTPA also presents specific challenges. A significant concern is the risk of over-diagnosing PE, which can lead to unnecessary anticoagulant treatment and increased healthcare costs. This issue arises from CTPA’s capacity to identify small or incidental emboli that may not have clinical significance. Distinguishing between clinically relevant and incidental emboli requires careful interpretation of the imaging findings by experienced radiologists (21, 22). Furthermore, CTPA involves exposure to ionizing radiation, which carries a small but cumulative risk of cancer, particularly in younger patients or those undergoing repeated imaging studies. Efforts to minimize radiation exposure, such as using lower-dose protocols and optimizing scan parameters, are essential to overcome this risk.

### 2.3 Current standard protocols for interpreting CTPA scans

Interpretation of CTPA scans involves a systematic evaluation of the pulmonary vasculature to identify the presence and extent of pulmonary embolism. Radiologists typically follow standardized protocols to ensure consistent and accurate interpretation of imaging findings. Key steps in interpreting CTPA scans include assessing the pulmonary arteries for filling defects, which indicate the presence of emboli, and evaluating the size and location of any detected emboli (23). The location of emboli, such as segmental or central arteries, within the pulmonary vasculature can reveal important details regarding the severity and prognosis of PE. In addition to diagnosing pulmonary embolism, CTPA scans may also reveal other lung abnormalities, such as pneumonia, lung nodules, or pleural effusions, which may have clinical significance and require further evaluation or management (24). Radiologists use various imaging features and scoring systems, such as the Qanadli or Miller scoring systems, to quantify the severity of pulmonary embolism and assess the risk of adverse outcomes. This information helps guide treatment decisions, such as initiating anticoagulant therapy or considering thrombolytic therapy in severe cases (23).

### 2.4 AI in CTPA-based in PE detection: the road so far

Despite the significant advances in the field, so far only two systematic reviews have provided comprehensive insights into the application of AI for PE detection through CTPA. Soffer et al. (25) conducted a meta-analysis examining deep learning applications for acute PE detection, analyzing seven studies that collectively evaluated 36,847 CTPA examinations. Their analysis revealed promising results, with pooled sensitivity and specificity of 0.88 and 0.86, respectively, demonstrating the potential effectiveness of deep learning algorithms in PE diagnosis. The studies included in their review predominantly utilized convolutional neural networks (CNNs) for direct embolism detection and classification.

In contrast, Abdulaal et al. (26) identified only five studies specifically addressing chronic PE and chronic thromboembolic pulmonary hypertension (CTEPH). Their review revealed diverse approaches to chronic PE detection, with studies focusing on various aspects such as lung parenchymal changes, PE classification, and hypoperfusion quantification. The reviewed studies showed variable performance metrics, though direct comparisons were challenging due to methodological differences and inconsistent reporting standards.

The disparity in research volume between acute and chronic PE detection is notable. While Soffer et al.’s review included studies with substantial datasets and more standardized approaches, Abdulaal et al.’s findings highlighted the limited research in chronic PE detection, suggesting a significant opportunity for expansion in this area. Both reviews identified similar limitations in their respective fields, including the predominance of retrospective studies and variable reporting quality across publications.

The systematic reviews also revealed interesting differences in methodological approaches. Studies reviewed by Soffer et al. primarily focused on direct embolism detection and classification, while those analyzed by Abdulaal et al. demonstrated more varied approaches, including the analysis of secondary features such as parenchymal changes and perfusion patterns. This difference reflects the distinct challenges in detecting chronic PE, which often presents with more subtle and varied radiological findings compared to acute cases.

Both systematic reviews emphasized critical needs in their respective fields. Soffer et al. highlighted the importance of prospective validation and standardized reporting, while Abdulaal et al. specifically noted the need for more consistent dataset reporting and improved model validation approaches. The systematic reviews collectively suggest that while acute PE detection algorithms have shown promising results and are gradually being implemented in clinical settings, the field of chronic PE detection remains in earlier stages of development.

The findings from these systematic reviews indicate a field in dynamic development, with varying levels of maturity between acute and chronic PE detection. As Abdulaal et al. note, the limited investigation of AI-based approaches for chronic PE represents an area of potential expansion for the field of AI in medical image interpretation. Meanwhile, Soffer et al.’s work suggests that deep learning models for acute PE detection are approaching clinical implementation, though further validation and standardization efforts are still needed.

Table 2 provides a side-by-side comparison of the key findings and characteristics from both systematic reviews, highlighting the current state of AI applications in both acute and chronic PE detection.

**Table 2:** Comparison of the key findings and characteristics from both systematic reviews, highlighting the current state of AI applications in both acute and chronic PE detection.

Characteristic	Soffer et al. (25)	Abdulaal et al. (26)
Focus	Acute PE Detection	Chronic PE/CTEPH Detection
Number of studies	7 studies	5 studies
Total sample size	36,847 CTPA studies	Variable across studies
Performance metrics	Sensitivity: 0.88 (95% CI: 0.803–0.927) Specificity: 0.86 (95% CI: 0.756–0.924)	AUC: 0.84–0.94 (varies by study)
AI methods	Predominantly CNNs	Deep learning approaches (CNNs)
Main applications	PE classification (present/absent) Embolism detection Segmentation	Lung parenchymal changes PE classification Hypoperfusion quantification 2D maximum intensity projection analysis
Study types	All retrospective	Predominantly retrospective
Key limitations	High risk of bias Limited prospective validation Need for external testing	Limited number of studies Inconsistent reporting Variable methodologies
Main findings	Consistent performance across studies Good diagnostic accuracy Ready for clinical validation	Emerging field Promising initial results Need for more research
Future directions	Need for prospective studies External validation Clinical implementation studies	Standardization of reporting Larger datasets Multi-center validation

Worth of note, the field of AI applications in pulmonary embolism detection reveals a striking scarcity of comprehensive reviews, as evidenced by the identification of only two systematic reviews (25, 26). This limited number of summary studies is particularly striking given the rapid advancement and growing implementation of AI technologies in medical imaging. The fact that these reviews are temporally separated and focus on different aspects of PE detection - acute and chronic presentations, respectively, - further emphasizes the need for an updated, comprehensive review that bridges these perspectives. Moreover, the most recent systematic review (26) specifically addresses chronic PE, leaving a three-year gap in the systematic analysis of acute PE detection advances since Soffer et al.’s work in 2021. This evident gap in the literature underscores the timeliness and significance of the present review, which aims to provide an up-to-date synthesis of the field’s progress, incorporating recent technological advances and emerging clinical applications that have yet to be systematically evaluated.

## 3 Role of AI in PE detection

### 3.1 Overview of AI technologies applied to medical imaging

Medical imaging plays a major role in identifying, planning treatment, and tracking many health disorders. Due to the progress in AI, specifically in deep learning algorithms, there has been a substantial increase in the use of AI technology for medical imaging. AI algorithms have proven remarkably adept at deciphering intricate medical images, enabling radiologists to identify patients more quickly and accurately (27). These AI technologies encompass various approaches, including convolutional neural networks (CNNs), generative adversarial networks (GANs), and recurrent neural networks (RNNs). In particular, CNNs have been used extensively for applications including feature extraction, classification, and image segmentation (28). RNNs are useful for tasks involving time-series imaging data because they are excellent at processing sequential data. AI algorithms have been used in several medical imaging methods, such as positron emission tomography (PET), ultrasound, CT, X-ray, and MRI. These technologies have shown promising results in detecting abnormalities, identifying patterns, and predicting patient outcomes based on imaging data (29, 30). Furthermore, AI-driven medical imaging solutions continuously evolve, incorporating advancements such as federated learning to maintain data privacy and transfer learning to leverage pre-trained models for specific medical imaging tasks (31). AI has the potential to completely transform medical imaging procedures as this field of study develops, leading to improved patient outcomes, tailored treatment plans, and more accurate diagnoses.

### 3.2 Application of AI in CTPA interpretation for PE detection

CTPA is a widely used imaging method for diagnosing PE and its interpretation typically depends on the knowledge of radiologists, a process that can be time-consuming and subject to variation. The use of AI technology has been on the rise in the interpretation of CTPA images for the identification of PE, providing numerous benefits (30). Deep learning algorithms, particularly CNNs, have demonstrated high accuracy in identifying and localizing pulmonary emboli on CTPA images. These AI systems can quickly analyze large volumes of image data, aiding radiologists in efficiently identifying and prioritizing cases requiring immediate attention (32).

In addition, AI-driven CTPA interpretation systems can help reduce interpretation errors and improve diagnostic accuracy by providing quantitative measurements and automated annotations of pulmonary emboli. By incorporating AI into the workflow, healthcare providers can expedite the diagnostic process, which improves patient outcomes and expedites the start of therapy (33). However, challenges such as robust validation, integration into existing clinical workflows, and addressing issues of interpretability and transparency remain important considerations in the widespread adoption of AI-assisted CTPA interpretation for pulmonary embolism detection.

**Figure 1:**
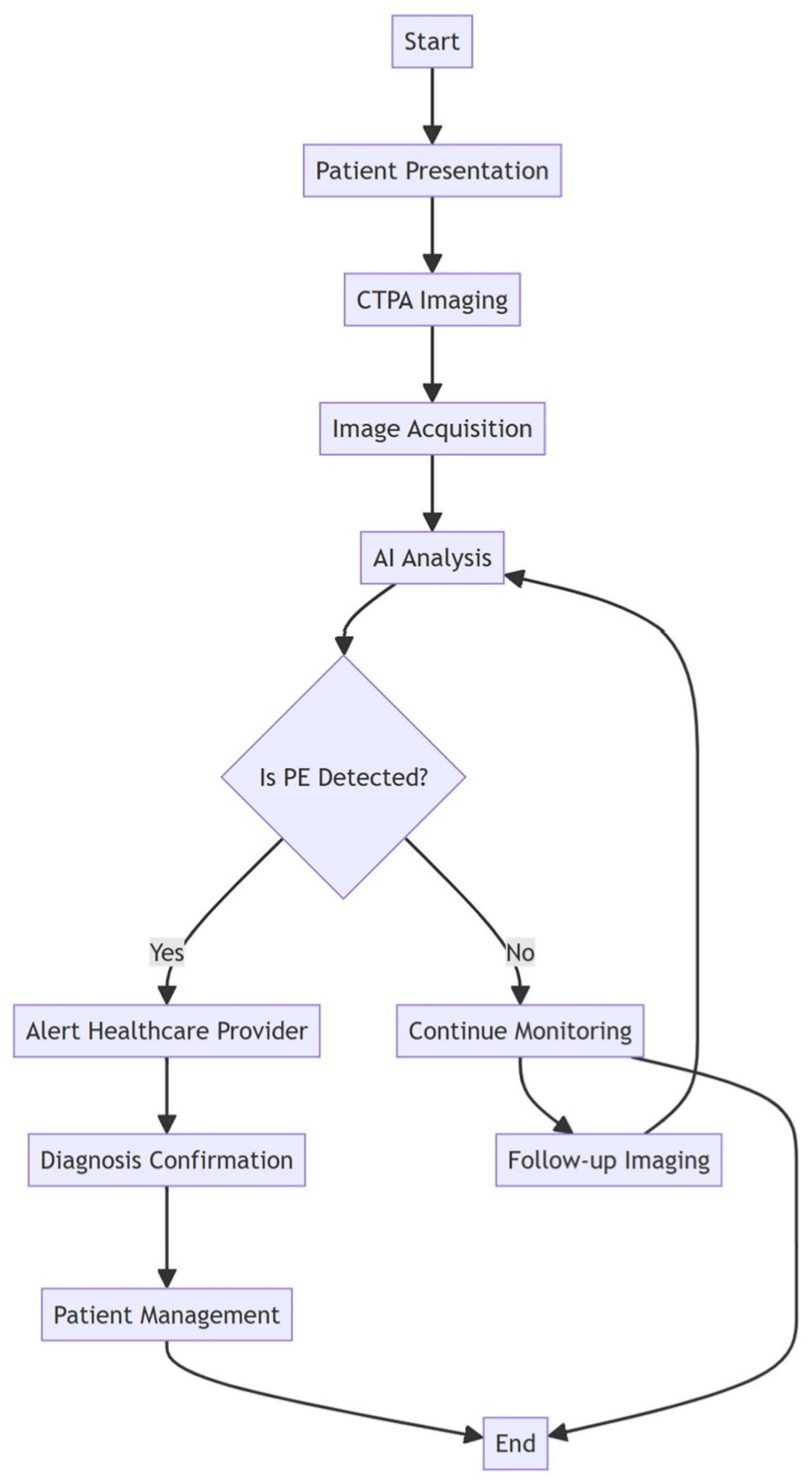
AI integration workflow in CTPA-based PE detection. Schematic representation of the systematic workflow for developing and validating an artificial intelligence (AI) system for pulmonary embolism (PE) detection on CT pulmonary angiography (CTPA). The process initiates with data acquisition and proceeds through three main phases: (1) Data Management: CTPA imaging data is systematically divided into training, validation, and test sets to ensure robust model development and unbiased evaluation. (2) Model Development: The AI model undergoes training using the training dataset, with validation data guiding parameter optimization. (3) Performance Assessment: The model’s diagnostic capability is evaluated through comprehensive metrics including sensitivity (ability to detect true PE cases), specificity (accuracy in identifying non-PE cases), positive and negative predictive values (PPV and NPV; reliability of positive and negative predictions), and area under the ROC curve (AUC-ROC; overall discriminative performance). The workflow incorporates a critical decision point (diamond) where performance metrics are compared against predetermined clinical thresholds. Models meeting these thresholds proceed to clinical deployment with continuous performance monitoring, while those requiring improvement undergo additional optimization cycles. Green circles denote workflow initiation and completion points, white rectangles represent process steps, yellow diamond indicates clinical decision point, light blue represents metrics calculation, and steel blue boxes show specific performance metrics. Arrows indicate process direction, with explicit “Yes/No” pathways at the clinical threshold assessment stage.

Figure 1. outlines the workflow from patient presentation to diagnosis confirmation and patient management, including the role of AI in analyzing CTPA images. The workflow begins with initial patient presentation and proceeds through several critical stages: (1) Clinical Phase: Following patient presentation, CTPA imaging is performed as the primary diagnostic tool. (2) Technical Phase: The process continues with image acquisition, where CTPA images are obtained and processed for AI analysis. (3) AI Implementation: The acquired images undergo automated AI analysis for PE detection. (4) Decision Point: The workflow branches based on AI findings - if PE is detected, healthcare providers are immediately alerted for further evaluation; if no PE is detected, the patient remains under monitoring. (5) Clinical Management: For positive cases, the diagnosis undergoes clinical confirmation followed by appropriate patient management protocols. For negative cases, the pathway includes follow-up imaging as needed, which feeds back into the AI analysis system, creating a continuous monitoring loop. The workflow concludes with either patient management for confirmed cases or continued monitoring for negative cases. Arrows indicate the directional flow of the process, with specific pathways marked for positive (“Yes”) and negative (“No”) AI findings. This integrated approach demonstrates the seamless incorporation of AI technology into the clinical decision-making process for PE detection.

### 3.3 Comparative analysis of AI-assisted diagnosis vs. traditional methods

The potential of AI-assisted diagnosis to enhance patient outcomes, diagnostic efficiency, and accuracy relative to traditional methods has gathered significant interest in its integration into medical practice. AI systems leverage machine learning algorithms to analyze medical data (including imaging studies, laboratory tests, and clinical notes) to assist healthcare providers in making informed decisions (34). One significant advantage of AI-assisted diagnosis is its ability to process vast amounts of data quickly and accurately, enabling the detection of subtle patterns and abnormalities that human practitioners may overlook. Additionally, AI algorithms can learn from large datasets and continuously improve their performance over time, leading to more reliable diagnoses (35). Furthermore, AI systems can provide decision support by generating differential diagnoses, recommending appropriate tests and treatments, and predicting patient outcomes based on clinical data. This can aid healthcare providers in developing personalized treatment plans tailored to individual patients’ needs.

**Figure 2:**
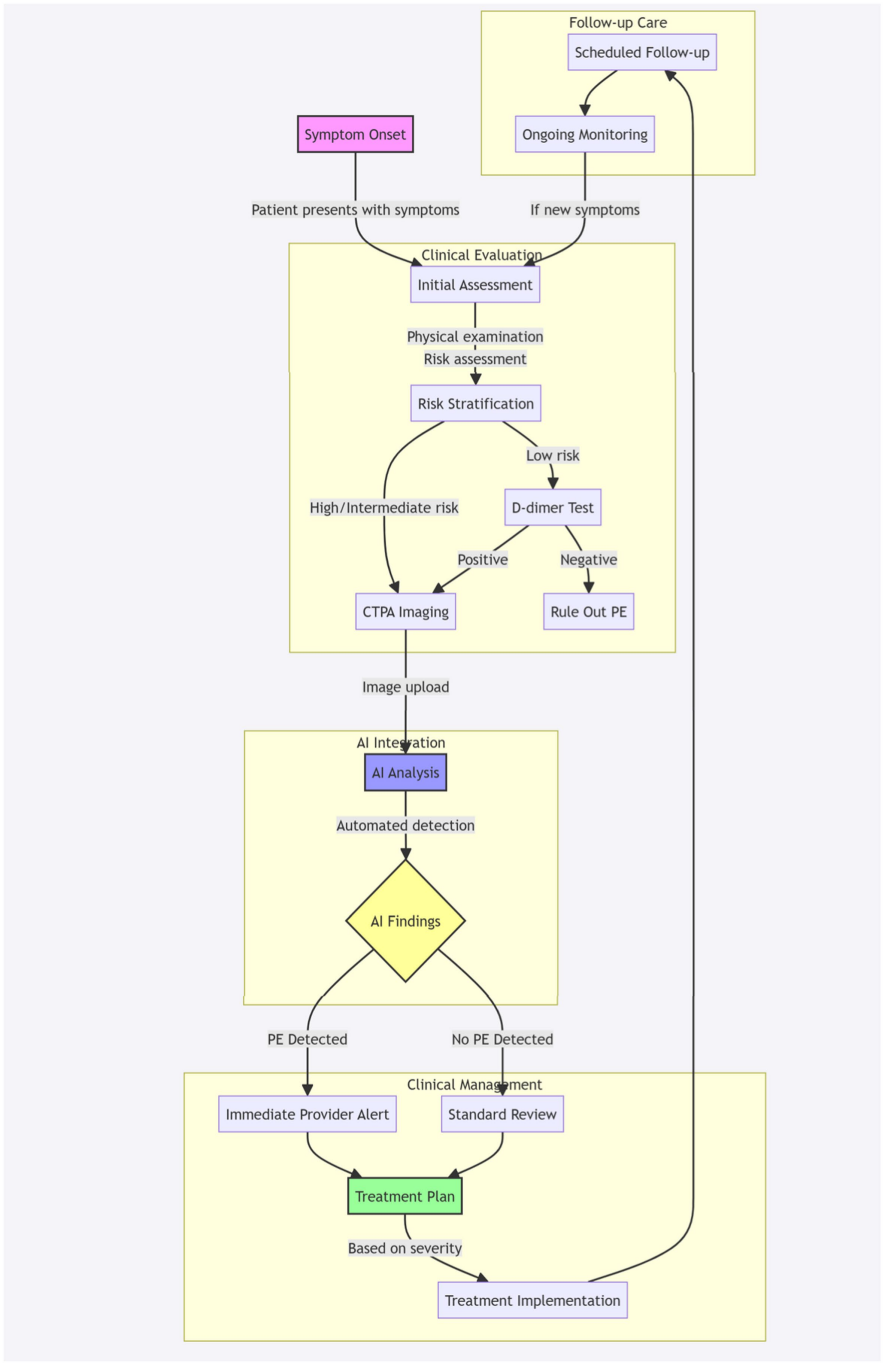
Patient journey with AI integration in PE detection. Workflow diagram illustrating the patient pathway from initial symptom presentation through diagnosis and management, incorporating AI-assisted PE detection. The process encompasses four key phases: clinical evaluation (risk stratification and initial testing), AI-integrated CTPA analysis, clinical management based on AI findings, and structured follow-up care. Pink node indicates entry point (symptom onset), blue node represents AI analysis, yellow node shows decision point, and green node indicates treatment planning. Arrows indicate process flow, with feedback loops for continuous monitoring.

Figure 2 illustrates the patient journey with AI integration in pulmonary embolism detection, outlining the workflow from initial symptom presentation through diagnosis and management. This comprehensive workflow diagram illustrates the patient’s journey through the PE diagnostic and management process with integrated AI support. The journey begins with symptom onset (pink node) and progresses through four main phases:

Clinical evaluation: Initial assessment includes physical examination and risk stratification, leading to either direct CTPA imaging for high/intermediate risk patients or D-dimer testing for low-risk patients.
AI integration: The acquired CTPA images undergo automated AI analysis (blue node), which processes the images to detect potential PE.
Clinical management: Based on AI findings (yellow decision node), the workflow branches into either immediate provider alerts for detected PE cases or standard review for negative cases, both leading to a comprehensive treatment plan (green node).
Follow-up care: The journey concludes with scheduled follow-up appointments and ongoing monitoring, with a feedback loop to initial assessment if new symptoms develop.

The workflow emphasizes the seamless integration of AI technology within the traditional clinical pathway, maintaining human oversight while leveraging automated detection capabilities. Colored nodes highlight critical decision points and process stages, while subgraphs organize the workflow into distinct clinical phases.

Nevertheless, it is important to recognize the constraints and difficulties associated with AI-supported diagnosis. These include concerns regarding algorithm bias, data quality and representativeness, interpretability of AI-generated recommendations, and potential impact on the physician-patient relationship. A comparative analysis between AI-assisted diagnosis and traditional methods should consider diagnostic accuracy, speed, cost-effectiveness, and impact on clinical workflow and patient care. While AI technologies promise to transform healthcare delivery, their successful integration into clinical practice requires careful validation, regulatory approval, and ongoing evaluation to ensure optimal performance and patient safety.

## 4 Performance and accuracy of AI in PE detection

The landscape of AI-based PE detection has evolved significantly over the last years, with several key studies demonstrating the potential of deep learning approaches in clinical settings. The sensitivity, specificity, and diagnostic accuracy are essential metrics for evaluating the performance of AI models in detecting PE from CTPA scans. Specificity evaluates the model’s capability to identify patients without PE, whereas sensitivity pertains to the model’s ability to classify patients with PE accurately (81). Diagnostic accuracy combines sensitivity and specificity to provide an overall measure of the model’s performance. Studies assessing AI models for PE detection have reported varying sensitivity and specificity rates, often ranging from 80% to over 95%. Higher sensitivity ensures fewer false negatives, reducing the chances of missing actual cases of PE, while higher specificity reduces false positives, avoiding unnecessffary treatment or further testing. Achieving a balance between sensitivity and specificity is of primary relevance for maximizing the diagnostic accuracy of AI models in PE detection (36).

**Figure 3:**
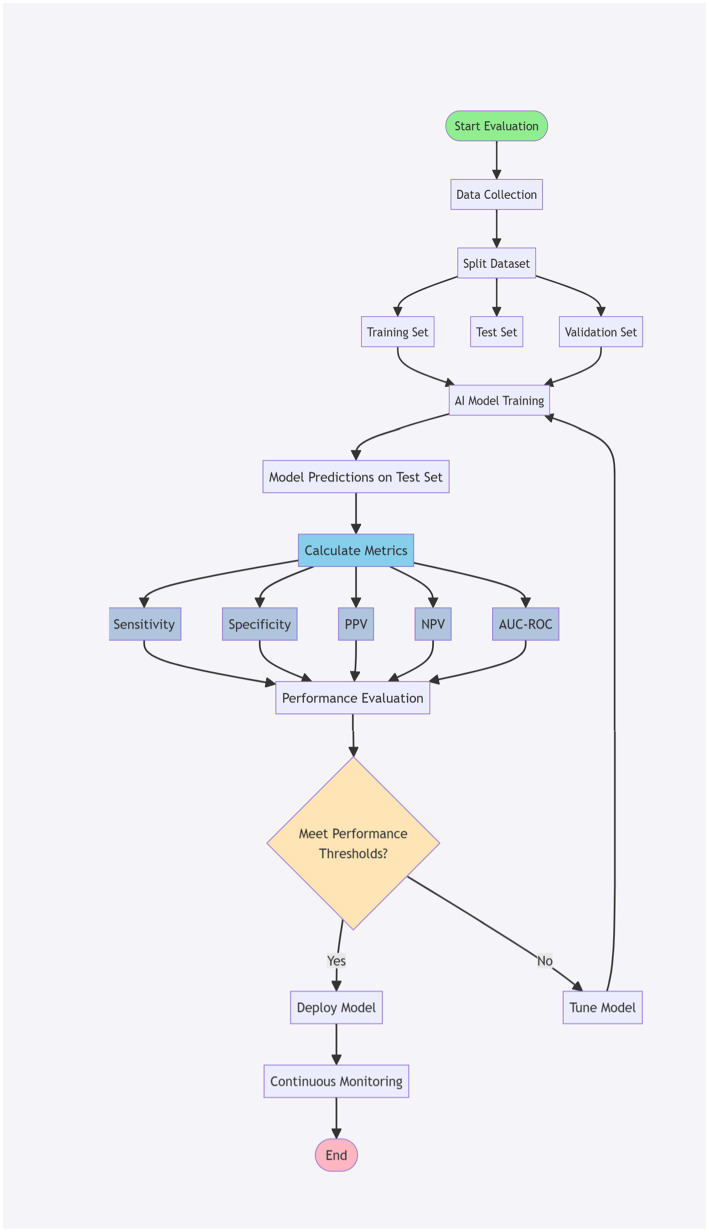
AI performance metrics workflow in PE detection. The flowchart demonstrates the systematic evaluation process of an AI system for PE detection. Starting with PE imaging data collection (white boxes), the workflow shows data division into training, validation, and test sets. The AI model undergoes training specifically for PE detection, followed by prediction generation. Performance assessment includes five key metrics (steel blue boxes): sensitivity, specificity, positive predictive value (PPV), negative predictive value (NPV), and area under the ROC curve (AUC-ROC). A clinical decision point (yellow diamond) determines whether the model meets established thresholds for clinical deployment. If standards are met, the model proceeds to clinical implementation with ongoing monitoring (right path); if not, it returns for further tuning (left path). Green circles indicate start and end points, while light blue boxes represent metrics calculation steps. Arrows indicate process flow, with specific “Yes/No” decision paths at the clinical threshold assessment point.

Figure 3 illustrates the AI performance metrics workflow in pulmonary embolism detection, outlining the systematic evaluation process of an AI system. This flowchart begins with the collection of PE imaging data and progresses through data division, model training, prediction generation, and performance assessment using key metrics such as sensitivity, specificity, PPV, NPV, and AUC-ROC.

### 4.1 Early validation studies

The initial wave of validation studies in AI-based PE detection established a strong foundation for future developments in this field. The pioneer word by Uddin et al. (37) conducted a comprehensive comparative analysis of different deep learning architectures. Their study evaluated three distinct approaches: a standard CNN, a residual network (ResNet), and a dense convolutional network (DenseNet). Using a dataset of 2,500 CTPA examinations, they found that the DenseNet architecture achieved the highest performance with an area under the curve (AUC) of 0.94, significantly outperforming traditional computer-aided detection systems.

Building upon these findings, Xie et al. (38) advanced the field by implementing a sophisticated convolutional neural network (CNN) architecture, trained on an even more extensive dataset of 3,000 CTPA scans. The algorithm achieved remarkable results with a sensitivity of 92.7% and specificity of 95.5%, comparable to expert radiologist performance. Notably, their system demonstrated particular strength in detecting peripheral PE cases, which are often challenging for human readers.

The scope of validation studies expanded significantly with Huang et al. (39)’s contribution, which conducted one of the first large-scale evaluations of deep learning for PE detection. Their study, utilizing 1,499 CTPA examinations, yielded promising results with a sensitivity of 82.4% and specificity of 81.8%. While these figures demonstrated the feasibility of automated PE detection, the study also provided valuable insights into the challenges of detecting small peripheral emboli, helping to shape future research directions.

In another significant study, Li et al. (40) developed and validated a deep learning algorithm using 3,635 CTPA examinations and was one of the first multi-center study in the field. Their system achieved an impressive area under the curve (AUC) of 0.95, with sensitivity and specificity of 92.7 and 95.5%, respectively. This work particularly demonstrated the importance of diverse training data in developing robust AI solutions.

### 4.2 Recent advances

Recent large-scale validation studies have demonstrated significant advances in AI performance for PE detection on CTPA, consistently show high diagnostic accuracy across different clinical settings, scanner manufacturers, and imaging protocols, with sensitivities ranging from 75–97% and specificities from 90–99.9%. For example, Kahraman et al. (41), evaluated an nnU-Net-based algorithm on 700 CTPA examinations, achieving 96.1% sensitivity and 94.6% specificity in internal validation, with maintained performance (98.4% sensitivity, 89.9% specificity) in external validation across 770 cases.

The robustness of AI performance has been demonstrated in challenging clinical scenarios. Topff et al. (42) and Zaazoue et al. (43) specifically assessed AI performance in COVID-19 patients, analyzing over 2,600 combined CTPA examinations. Their studies showed maintained high accuracy (sensitivity: 91.6–93.2%, specificity: 99.6–99.7%) despite varying degrees of COVID-19 parenchymal involvement. These findings were particularly significant given the increased complexity of PE detection in the context of COVID-19-related lung changes.

Recent studies have also expanded beyond simple detection capabilities. Djahnine et al. (44) demonstrated the feasibility of automated severity quantification using the Qanadli score and RV/LV ratio measurements (*R*^2^ = 0.717 and 0.723 respectively). This advancement in automated severity assessment represents a significant step toward comprehensive AI assistance in PE management. Similarly, Baeza et al. (45) explored novel applications in Q-SPECT/CT analysis without ventilation imaging, achieving 75.1% sensitivity and 98.2% specificity, opening new possibilities for PE detection in alternative imaging modalities.

Importantly, several studies have demonstrated AI’s potential to reduce radiologist miss rates. Ayobi et al. (46) showed that AI could detect 76% of PE cases initially missed in clinical reports, potentially reducing radiologist miss rates from 15.6 to 3.8%. However, researchers consistently emphasize AI’s role as complementary to radiologists rather than a replacement, with studies showing improved radiologist confidence and diagnostic accuracy when using AI as an assistive tool, which is discussed section 4.3. In addition, multi-center studies have provided strong evidence for AI’s generalizability, as discussed in section 4.4.

### 4.3 Comparison with human radiologists and traditional image interpretation methods

The performance and accuracy of AI algorithms in detecting PE from CTPA scans have been extensively compared with human radiologists and traditional image interpretation methods (47). Multiple studies have shown that AI algorithms may achieve similar or even better results than human radiologists in terms of sensitivity, specificity, and diagnostic accuracy. For example, a study by Liu et al. (33) demonstrated that a deep-learning algorithm outperformed radiologists in detecting PE from CTPA scans with higher sensitivity and specificity (48). Additionally, AI algorithms have shown the potential to reduce interpretation time and improve workflow efficiency compared to traditional methods. However, challenges such as interpretability, generalizability, and integration into clinical practice remain significant difficulty for widespread adoption (49).

Another significant contribution to this field comes from the recent study by Langius-Wiffen et al. (50), who conducted a large-scale retrospective study analyzing 3,316 CTPA scans using an FDA-approved and CE-marked AI algorithm. Their findings demonstrated that the AI algorithm achieved significantly higher diagnostic accuracy compared to radiologists, with sensitivity of 96.8% versus 91.6% and specificity of 99.9% versus 99.7%. Notably, the AI system missed only 23 PE cases compared to 60 missed by radiologists, while producing just 2 false positives compared to 9 by radiologists.

These results align with earlier findings by Liu et al. (33), who demonstrated that deep-learning algorithms could outperform radiologists in detecting PE from CTPA scans with higher sensitivity and specificity (48). However, Langius-Wiffen et al. (51) emphasize that standalone use of AI algorithms is currently not warranted. Their study highlights that reading CTPAs requires analysis beyond just PE detection, as radiologists must identify other relevant pathologies. Current algorithms focused solely on PE detection may miss other clinically relevant findings, such as thrombus in the right atrial appendage, signs of significant pulmonary hypertension, right-ventricular pressure overload, and various additional findings of infectious, oncological, or cardiovascular etiology. On this aspect, Ben Cheikh et al. (34) conducted a significant multicenter study evaluating an FDA-approved and CE-marked AI algorithm for PE detection on CTPA across 1,202 patients. The AI demonstrated higher sensitivity (92.6% vs. 90.0%) and negative predictive value (98.6% vs. 98.1%) compared to radiologists, though lower specificity (95.8% vs. 99.1%) and positive predictive value (80.4% vs. 95.0%). The AI detected 19 PEs missed by radiologists (approximately 1 PE per 63 CTPAs) and proved particularly valuable for poor-quality examinations. While AI implementation increased radiologists’ diagnostic confidence, with 72.2% reporting positive impact on their practice, it also slightly increased interpretation time by about 1 min per case. The authors concluded that AI serves best as a complementary tool to augment radiologist performance rather than a replacement, particularly acting as a “safety net” in emergency radiology practice.

The path forward lie in implementing AI as an assistive tool rather than a replacement for radiologist expertise. Further research and validation, particularly through clinical utility studies, are needed to establish AI’s reliability and clinical utility in PE detection compared to human radiologists and traditional image interpretation methods. This approach would leverage the strengths of both AI and human expertise, potentially leading to improved patient outcomes through enhanced diagnostic accuracy and efficiency.

### 4.4 Large-scale multi-center studies

Recent advances in AI applications for PE detection have demonstrated significant progress through several landmark multi-center studies. These studies have collectively advanced our understanding of both the technical capabilities and practical implementation challenges of AI in clinical settings.

The MP-Net study represents a significant advancement in privacy-preserving frameworks for medical image segmentation (52). Using 7,279 CTPA studies from five different clinical sites, it was achieved remarkable performance metrics with a dice score of 91.8% and sensitivity of 98.0%. This study particularly addressed one of the fundamental challenges in multi-center collaboration: data privacy. The innovative network architecture enables secure data sharing between centers while maintaining patient confidentiality, establishing a potential blueprint for future collaborative efforts.

Grenier et al. (53) validated their algorithm across 228 U.S. clinical sites, maintaining consistent performance (91.4% sensitivity, 91.5% specificity) regardless of scanner type or technical parameters. Ben Cheikh et al. (34) further supported these findings through their multi-center study of 1,202 patients, where AI detected 19 additional PEs missed in clinical practice.

Condrea et al. (54) introduced a novel architectural approach to PE detection. Working with the RSNA PE CT multi-center dataset, this study achieved an F1 score of 91.0% through its innovative two-phase detection approach. The incorporation of anatomical awareness into the detection pipeline represents a significant step toward more sophisticated and clinically relevant AI systems.

A broader perspective on pulmonary vasculature analysis emerged from Chu et al. (55), which examined 11,784 participants across six Chinese medical centers. Artery–vein segmentation is a relevant diagnostic indication for pulmonary and cardiovascular diseases such as pulmonary embolisms and pulmonary arterial hypertension. This comprehensive investigation revealed previously undocumented associations between pulmonary vascular anatomy and demographic characteristics. This study not only demonstrated the feasibility of contrast-agent-free segmentation but also revealed important demographic associations with vessel anatomy. The scale and comprehensive nature of this study provide valuable insights into population-level variations in pulmonary vasculature.

Condrea et al. (54) approached the challenge from a different angle, developing a weakly supervised learning framework for PE segmentation. Using the RSPECT dataset, the researchers achieved an F1 score of 71.6%, demonstrating the potential of generating detailed pixel-level annotations from more readily available image-level labels. This approach could significantly reduce the annotation burden in future studies while maintaining clinical utility.

In contrast to these large-scale studies, Kim et al. (56) focused on a specific clinical application: predicting PE and reducing unnecessary CTPA scans in gastrointestinal cancer patients. Despite its smaller scale of 585 patients from two hospitals, the study achieved notable AUROC values of 0.736 and 0.669 for internal and external validation, respectively. This research highlights the importance of targeted applications in specific patient populations.

These studies collectively reveal several emerging trends in the field. First, there is a clear movement toward larger, more diverse datasets that better represent real-world patient populations. Second, privacy-preserving methods are becoming increasingly central to multi-center collaboration, with new architectures specifically designed to address these concerns. Third, there is growing emphasis on clinical integration and practical utility, with many studies focusing on reducing unnecessary imaging while maintaining diagnostic accuracy.

Looking forward, the field is moving toward more sophisticated, clinically integrated solutions that can be used across multiple centers while maintaining patient privacy and data security. Future developments will likely focus on standardization of protocols and reporting, enhancement of privacy-preserving methods, and seamless integration into clinical workflows. The success of these large-scale multi-center studies suggests that AI-based PE detection is maturing into a clinically viable tool, though continued validation and refinement will be essential for widespread adoption.

## 5 Advantages and benefits of AI in PE detection

### 5.1 Enhanced diagnostic efficiency and processing speed

AI demonstrates significant advantages in PE detection through its computational efficiency and rapid processing capabilities. While conventional PE diagnosis relies on manual interpretation of radiological images and clinical data by specialists, AI algorithms leverage parallel processing to analyze vast datasets simultaneously. Contemporary deep learning architectures, particularly CNNs and Vision Transformers, demonstrate remarkable efficiency in processing CTPA images, achieving analysis speeds of hundreds of images per minute (57–59).

The acceleration of diagnostic processes directly correlates with reduced time-to-treatment intervals, potentially improving clinical outcomes through earlier intervention. Studies indicate that AI-assisted PE detection can reduce interpretation times by 30–40% compared to traditional methods (39). These systems excel in rapid identification of both central and peripheral emboli, with particular effectiveness in detecting subsegmental PE, which traditionally presents diagnostic challenges (60). Furthermore, modern AI algorithms incorporate automated preprocessing techniques, including image normalization and artifact reduction, enhancing the quality and reliability of rapid analysis.

### 5.2 Reduction in diagnostic errors and false negatives

The implementation of AI systems in PE detection presents a quantifiable reduction in diagnostic errors and missed cases. Despite high expertise levels, human interpretation of radiological images remains inherently subjective and vulnerable to cognitive biases. New studies indicates that radiologist accuracy in PE detection varies between 67 and 84% depending on experience and image quality (61, 62). Furthermore, diagnostic accuracy can be compromised by factors such as radiologist fatigue and high case volumes, with error rates increasing by up to 12% during extended reading sessions (60).

AI algorithms demonstrate consistent performance in detecting subtle radiological abnormalities, maintaining uniform sensitivity across large datasets. Through iterative learning processes and continuous algorithm refinement, these systems achieve sensitivity rates exceeding 90% and specificity rates of 85–95% (25). Modern deep learning models incorporate attention mechanisms and feature extraction techniques that enable:

- Detection of subtle filling defects in peripheral vessels.
- Accurate quantification of clot burden.
- Identification of associated cardiovascular complications.
- Assessment of right ventricular strain patterns.

The standardization in diagnostic approach contributes to enhanced patient safety and more reliable treatment planning protocols.

### 5.3 Optimization of clinical workflow and decision support

Modern AI-enabled workflow systems have revolutionized the diagnostic process through sophisticated automated triage and prioritization mechanisms. These systems implement real-time case prioritization based on PE probability, while simultaneously managing intelligent worklists and generating automatic notifications for critical findings. The continuous integration with hospital information systems further enhances the overall efficiency of the diagnostic workflow.

The advanced decision support capabilities of contemporary AI systems extend beyond basic image analysis. These systems perform comprehensive quantitative analyses of clot burden using standardized metrics and automated calculations of right ventricle/left ventricle (RV/LV) ratios (63). Furthermore, they facilitate sophisticated risk stratification based on imaging biomarkers and integrate clinical parameters with imaging findings, providing radiologists with a holistic view of each case (64–66). This integration of multiple data points enables more informed clinical decision-making and supports the development of personalized treatment strategies.

Quality assurance represents another critical aspect of AI-enhanced workflows. Through continuous performance monitoring and automated quality control of image acquisition, these systems maintain consistent diagnostic standards. The implementation of standardized reporting templates, coupled with comprehensive audit trail functionality, ensures accountability and facilitates ongoing quality improvement initiatives (67).

The dynamic nature of AI systems in PE detection is particularly evident in their capacity for continuous learning and improvement. Regular model updates incorporate new data and feedback from radiologists, allowing the systems to adapt to local practice patterns and emerging clinical guidelines (68). This adaptive capability ensures that the AI systems remain current with evolving medical knowledge and practice standards. The implementation of these advanced AI systems has demonstrated remarkable improvements in operational efficiency, with research indicating substantial reductions in radiologist reading time and preliminary report generation time (69).

The synergistic relationship between AI systems and clinical expertise has transformed the traditional diagnostic pathway (70). Enhanced communication between clinical teams, coupled with streamlined workflows, has led to more efficient healthcare delivery and improved patient outcomes. This optimization of clinical workflows through AI integration represents a significant advancement in radiological practice, establishing new standards for diagnostic efficiency and accuracy in PE detection. The continuous evolution of these systems, driven by ongoing technological advances and clinical feedback, suggests that further improvements in workflow optimization and decision support capabilities will continue to emerge, further enhancing the role of AI in PE detection and management (22, 71).

**Figure 4:**
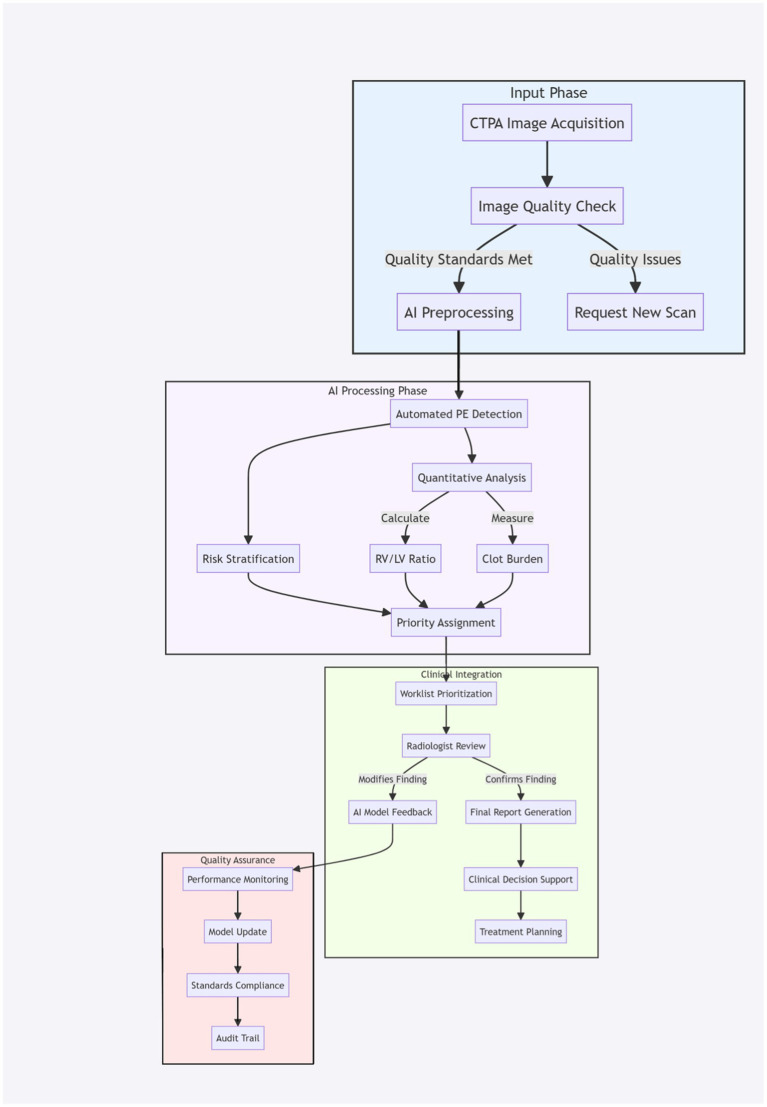
AI-enhanced workflow for pulmonary embolism detection and management. The workflow is divided into four major phases (indicated by colored sections): Input Phase (blue), AI Processing Phase (purple), Clinical Integration (green), and Quality Assurance (red). Arrows indicate the direction of workflow progression. Boxes represent individual processes or actions. Parallel processes are shown at the same horizontal level. Feedback loops demonstrate the continuous improvement aspects of the system.

Figure 4 illustrates the comprehensive workflow of AI-enhanced PE detection and management, demonstrating the integration of AI with clinical practice. The workflow comprises four interconnected phases that ensure efficient diagnosis while maintaining quality standards and enabling continuous system improvement.

The Input Phase (blue) initiates with CTPA image acquisition, followed by automated quality assessment. This phase incorporates a quality control feedback loop, ensuring that only images meeting predetermined quality standards proceed to AI analysis. Substandard images are flagged for repeat acquisition, maintaining high diagnostic standards from the outset.

The AI Processing Phase (purple) represents the core analytical components of the system. Here, the AI simultaneously performs multiple analyses: automated PE detection, quantitative measurements including right ventricle/left ventricle (RV/LV) ratio calculation, and clot burden assessment. These parallel processes contribute to an automated risk stratification system that determines case priority, enabling efficient resource allocation and rapid identification of high-risk cases.

The Clinical Integration phase (green) demonstrates the crucial interaction between AI and clinical expertise. Cases are automatically prioritized in the radiologist’s worklist based on AI findings and risk assessment. This phase emphasizes the supervisory role of radiologists, who can either confirm or modify AI findings. Confirmed cases proceed to standardized report generation and clinical decision support, while modified cases generate valuable feedback for system improvement.

The Quality Assurance phase (red) completes the cycle through continuous performance monitoring and system refinement. This phase is critical for maintaining diagnostic accuracy and ensuring ongoing system improvement. The feedback loop from radiologist modifications contributes to model updates, while maintaining compliance with clinical standards and generating comprehensive audit trails for quality control.

The bidirectional arrows between phases highlight the dynamic nature of the workflow, where information flows not only forward through the diagnostic process but also backward through feedback mechanisms. This design ensures that the system continuously learns and adapts to local practice patterns while maintaining high diagnostic standards.

This workflow represents a significant advancement over traditional diagnostic pathways by optimizing resource utilization, standardizing diagnostic approaches, and maintaining quality control through continuous feedback and improvement cycles. The integration of AI technology with clinical expertise creates a synergistic system that enhances both efficiency and accuracy in PE detection and management.

The workflow diagram provides a clear visualization of how modern AI systems can be effectively integrated into clinical practice while maintaining the essential role of clinical expertise and oversight.

## 6 Limitations and challenges of AI in PE detection

### 6.1 The SFR 2022 AI data challenge

The SFR 2022 AI data challenge represents a significant milestone in understanding the complexities of implementing AI for PE detection and assessment (72). While the initiative successfully brought together multiple institutions and demonstrated promising results, it also revealed several interconnected challenges that merit careful consideration for future developments in this field.

#### 6.1.1 Data complexity and annotation challenges

A fundamental limitation emerged from the study’s data structure and annotation process. Although the challenge accumulated 1,268 CT examinations from 16 French centers (a substantial achievement in multicenter collaboration) this dataset reveals inherent limitations in current AI development approaches. The absence of detailed image-level annotations, particularly crucial for complex measurements like Qanadli’s score, highlights a critical gap between clinical practice and AI training requirements. This limitation becomes particularly significant when considering that Qanadli’s score calculation demands precise understanding of pulmonary arterial anatomy and embolism location, information that was only provided as a final numerical score rather than detailed anatomical annotations.

#### 6.1.2 Methodological constraints and clinical reality

The challenge’s structure, while well-organized, exposed several methodological constraints that reflect broader issues in AI development for medical imaging. The separation of the three tasks—PE detection, RV/LV ratio measurement, and Qanadli’s score calculation—into independent evaluations, though practical for the competition, may not align with clinical reality where these parameters are inherently interconnected. The winning team’s overall score of 0.784 demonstrates both the promise and limitations of current AI approaches, suggesting that while automated analysis is feasible, achieving clinical-grade accuracy across all tasks remains challenging.

#### 6.1.3 Generalizability and external validity

A more subtle but crucial limitation emerges from the study’s geographical and institutional constraints. Despite its multicenter nature, the dataset was exclusively sourced from French institutions, potentially limiting the AI models’ generalizability to different healthcare systems, patient populations, and clinical protocols. This limitation is particularly relevant given that PE presentation and imaging characteristics may vary across different populations and healthcare settings. The challenge’s approach of allowing teams to use external datasets for training partially addresses this issue but also introduces questions about standardization and validation of these supplementary data sources.

#### 6.1.4 Technical implementation and workflow integration

The challenge revealed significant technical hurdles in implementing AI solutions for PE assessment. The competition’s design, which provided data in three batches, highlighted the importance of robust and adaptable AI systems. However, it also exposed the difficulties in creating solutions that can seamlessly integrate into various clinical workflows while maintaining consistent performance across different CT protocols and equipment. The minimal preprocessing approach adopted by the challenge organizers, while providing flexibility to competitors, emphasizes the need for robust standardization methods in real-world applications.

#### 6.1.5 Clinical validation and safety considerations

Perhaps the most critical limitation relates to clinical validation and safety considerations. The evaluation metrics chosen—AUC for detection and R^2^ for regression tasks—while statistically sound, may not fully capture the clinical implications of AI performance. The challenge’s structure did not fully address how these systems would perform under real-world conditions, particularly in emergency settings where time constraints and clinical pressure are significant factors. Furthermore, the critical issue of false negatives in PE detection, which carries significant patient safety implications, requires more comprehensive evaluation frameworks than those typically used in research settings.

### 6.2 Additional challenges and limitations

In addition to the limitations identified in the SFR 2022 AI challenge study, several critical challenges need to be addressed for successful implementation of AI-based PE detection systems. These challenges span multiple domains and require careful consideration for future development.

#### 6.2.1 AI models for PE detection

Training AI models for PE detection faces several data limitations and challenges. Firstly, acquiring high-quality labeled data for training AI algorithms can be difficult. Annotated medical imaging data, such as CTPA scans, require expert radiologists to accurately label PE instances, which can be time-consuming and costly. Additionally, the availability of diverse and representative datasets encompassing various patient demographics, comorbidities, and imaging artifacts is decisive to ensure the robustness and generalizability of AI models (73). Nevertheless, acquiring such extensive datasets might present difficulties due to privacy considerations, limitations on data access, and the fragmented distribution of healthcare data among many institutions and systems. Furthermore, data imbalance, where negative cases significantly outnumber positive cases of PE, can lead to biased model performance and reduced sensitivity in detecting rare or subtle PE instances (82). Addressing these data limitations and challenges requires collaborative efforts among healthcare providers, researchers, and policymakers to establish standardized data-sharing protocols, enhance data quality and annotation processes, and promote transparency and accountability in AI model development and evaluation.

#### 6.2.2 Interpretation of complex CTPA findings beyond simple emboli detection

While AI algorithms have shown promising results in detecting PE on CTPA scans, interpreting complex findings beyond simple emboli detection presents significant challenges. CTPA images often contain anatomical structures, physiological variations, and imaging artifacts that can confound AI algorithms’ interpretation and lead to false positives or negatives (39). Furthermore, distinguishing between acute and chronic PE, identifying alternative diagnoses mimicking PE, and assessing the clinical significance of detected emboli require nuanced clinical judgment and domain expertise that may not be fully captured by AI systems alone (33). In addition, integrating contextual information from patients’ clinical histories, laboratory tests, and other diagnostic modalities is essential for accurate diagnosis and appropriate patient management. Still, this complexity presents additional challenges for AI algorithms in terms of effective processing and integration. To address these issues, it is essential to develop AI models that use techniques for integrating diverse data types, implement mechanisms for contextual understanding, and offer interactive tools to assist radiologists and clinicians in accurately and efficiently interpreting complex CTPA findings.

#### 6.2.3 Real-time performance and resource requirements

The implementation of AI systems for PE detection presents complex challenges in computational infrastructure and performance optimization. Healthcare institutions must carefully consider the substantial computational resources required for real-time analysis of medical imaging data. Modern AI systems demand high-performance GPU configurations capable of processing multiple CT scans simultaneously while maintaining rapid response times essential for emergency care scenarios.

The computational infrastructure must be designed to handle varying workloads efficiently, particularly during peak usage periods when multiple departments may require simultaneous access. This necessitates sophisticated load balancing mechanisms and resource allocation strategies. Storage systems must accommodate not only the large volumes of imaging data but also maintain rapid access capabilities through efficient cache management and data retrieval protocols.

Network infrastructure plays a crucial role in system performance, as the movement of large imaging datasets between storage, processing units, and viewing stations requires substantial bandwidth. Organizations must carefully balance the trade-offs between cloud-based and on-premises solutions, considering factors such as data security, access speed, and scalability requirements. The system architecture must incorporate redundancy measures and failover capabilities to ensure continuous availability, particularly critical in emergency medicine contexts.

#### 6.2.4 Standardization and interoperability

Standardization and interoperability represent fundamental challenges in the widespread adoption of AI systems for PE detection. The healthcare environment typically involves multiple vendors, various imaging equipment manufacturers, and diverse IT systems, all of which must work seamlessly together. Implementation of standardized DICOM formatting for AI results becomes crucial, ensuring that findings can be consistently interpreted across different platforms and institutions.

Integration protocols must adhere to established healthcare standards such as HL7/FHIR, enabling smooth communication between AI systems and existing healthcare infrastructure. This standardization extends beyond mere technical compatibility to encompass structured reporting templates and common annotation formats, ensuring consistency in how AI findings are documented and communicated across different healthcare settings (74).

Version control and update management present additional challenges in maintaining system consistency. Healthcare institutions must establish robust protocols for managing software updates, ensuring backward compatibility, and maintaining detailed documentation of system changes. This becomes particularly complex in multi-vendor environments where different components may update at different intervals.

#### 6.2.5 Quality assurance and system monitoring

Quality assurance in AI-based PE detection systems requires comprehensive monitoring frameworks that track both technical performance and clinical impact. Continuous monitoring of system performance must go beyond simple accuracy metrics to include detailed analysis of false positive and negative rates, processing time variations, and system availability statistics. This monitoring should be automated where possible, with clear protocols for identifying and addressing performance degradation.

Error management systems must be sophisticated enough to detect both obvious failures and subtle degradation in performance. Root cause analysis procedures should be established to investigate significant errors, with clear protocols for implementing corrective actions. The quality assurance framework should also include regular assessment of clinical outcomes, evaluating how AI system performance correlates with patient outcomes and treatment decisions.

System maintenance becomes a critical component of quality assurance, requiring regular optimization routines and health checks. Security updates must be carefully managed to maintain system integrity while ensuring minimal disruption to clinical workflows. Database maintenance procedures should be implemented to manage the growing volume of imaging data while maintaining rapid access capabilities.

#### 6.2.6 Educational and training requirements

The successful implementation of AI systems for PE detection necessitates comprehensive educational programs that address both technical and clinical aspects of the technology. Initial training programs must provide healthcare professionals with a fundamental understanding of AI concepts, including its capabilities and limitations. This knowledge base helps ensure appropriate use of the technology and maintains clinical judgment in decision-making processes.

Ongoing education becomes essential as systems evolve and capabilities expand. Regular updates to training materials should reflect system improvements and address any newly identified limitations or considerations. Clinical staff must understand not only how to operate the system but also how to interpret results in the context of their clinical expertise.

Professional development programs should include practical scenarios and case studies that demonstrate both typical and edge cases. These programs should emphasize the complementary nature of AI systems, reinforcing that they are tools to enhance, rather than replace, clinical judgment. Special attention should be given to training in error recognition and appropriate escalation procedures when system results appear inconsistent with clinical findings.

The educational framework must also address the broader implications of AI implementation, including changes to workflow patterns, documentation requirements, and communication protocols. This comprehensive approach ensures that all stakeholders understand their roles in maintaining system effectiveness and patient safety. Regular assessment of training effectiveness helps identify areas requiring additional focus or modification, ensuring that educational programs evolve with technological advances and changing clinical needs.

Advancements in AI interpretability, particularly through the development of explainable AI, help alleviate clinician skepticism surrounding AI adoption in healthcare. Providing clear and understandable explanations for AI-generated recommendations and predictions enables clinicians to grasp the rationale behind the system’s outputs. This transparency fosters trust and confidence in the technology, as healthcare professionals can better assess the reliability and relevance of AI insights in their clinical decision-making processes. Furthermore, incorporating explainability into training programs equips clinicians with the tools to critically evaluate AI suggestions, ensuring they can integrate AI findings with their own clinical expertise. Bridging the gap between complex AI algorithms and clinical practice, explainable AI enhances the perceived value of AI systems and reinforces the notion that these technologies are designed to support, rather than supplant, clinical judgment.

#### 6.2.7 Regulatory and ethical considerations in AI adoption for medical diagnosis

Adopting AI for medical diagnosis, particularly in the detection of pulmonary embolism (PE), raises several regulatory and ethical considerations that must be meticulously addressed to ensure patient safety, data privacy, and healthcare equity. Regulatory agencies, such as the Food and Drug Administration (FDA), play a crucial role in overseeing the safety and effectiveness of AI-based medical devices. This oversight necessitates rigorous validation studies to ensure that AI systems are thoroughly tested for accuracy and reliability, along with transparent reporting of algorithm performance metrics. Continuous monitoring of real-world outcomes is also essential to assess ongoing efficacy (33).

Compliance with medical device regulations, data protection laws (e.g., HIPAA), and ethical principles (e.g., beneficence, autonomy, justice) requires comprehensive risk assessment and the establishment of robust governance frameworks. Engaging stakeholders, including healthcare institutions, industry partners, and regulatory bodies, in the decision-making process is vital to fostering a collaborative environment that prioritizes patient safety and ethical standards (30).

A significant ethical concern in AI adoption is the potential for bias, particularly against populations that are underrepresented in training datasets. Such biases can lead to inequitable clinical outcomes, as AI systems may perform poorly for certain demographic groups, resulting in misdiagnoses or inadequate treatment recommendations. This not only jeopardizes patient safety but can also erode trust among patients from these populations, further exacerbating existing health disparities. To mitigate these biases, it is critical to actively seek diverse populations for inclusion in training datasets, ensuring that AI algorithms can generalize effectively across different demographic groups. Regular audits of AI systems should be implemented to identify and address biases in algorithm performance. Additionally, engaging with community representatives and advocacy groups can provide valuable insights into the needs and concerns of underrepresented populations, ensuring their voices are included in the development and evaluation processes of AI technologies.

Collaborative initiatives among policymakers, researchers, industry leaders, and patient advocates are essential for developing regulatory guidelines, creating ethical frameworks, and establishing best practices that encourage responsible AI adoption in medical diagnosis. On addressing these regulatory and ethical considerations, particularly concerning AI bias, stakeholders can promote the safe and effective integration of AI technologies in medical diagnostics, ultimately enhancing patient care and outcomes.

## 7 Future directions and research opportunities

### 7.1 Emerging trends and developments in AI for PE detection

As technology progresses, AI plays an increasingly major role in detecting PE. Emerging trends indicate a shift toward using deep learning algorithms, particularly CNNs, for more accurate and efficient diagnosis. These CNNs can analyze vast amounts of medical imaging data, such as CTPA scans, to identify subtle signs of PE that may be overlooked by human observers (25). Natural language processing (NLP) advancements enable AI systems to extract relevant information from clinical notes and reports, facilitating faster and more accurate diagnosis (25, 34). Integrating AI with wearable devices and remote monitoring technologies holds promise for real-time detection and early intervention in patients at risk of PE, thus improving outcomes and reducing mortality rates.

### 7.2 Potential integration of AI with other diagnostic methods for comprehensive evaluation

Integrating AI with other diagnostic modalities presents an exciting opportunity for comprehensive assessment in PE detection. Integrating AI algorithms with imaging modalities like ultrasound and MRI can offer a comprehensive perspective on the patient’s state, improving diagnostic precision and minimizing the chances of incorrect diagnosis (75, 76). Similarly, incorporating clinical data into AI models, including laboratory tests and patient history, can improve their predictive capabilities. Clinicians may acquire a more comprehensive understanding of PE pathology by using a multi-modal approach, which can result in more informed treatment decisions and better patient outcomes. Furthermore, decentralized PE diagnosis may be possible through the integration of AI with point-of-care testing tools and mobile health applications, especially in areas with low resources and restricted access to specialized medical facilities.

### 7.3 Areas for future research and refinement of AI algorithms in PE detection

Despite significant advancements highlighted in this review, several areas remain for future research and refinement of AI algorithms in PE detection. One important area is the development of interpretable AI models, which boost confidence and make it easier to incorporate AI into clinical practice by providing physicians insights into the decision-making process. The robustness and generalizability of AI algorithms across diverse patient populations and imaging platforms also need to be further investigated to ensure reliable performance in real-world settings. Furthermore, continued efforts are required to enhance the sensitivity and specificity of AI models, particularly in detecting small or subsegmental emboli that may be clinically significant but challenging to identify. AI’s ethical and regulatory implications in PE detection also deserve attention, including data privacy, transparency, and accountability, warrant careful consideration to ensure responsible deployment and adoption. Collaborative research efforts among clinicians, data scientists, and regulatory authorities are essential to address these challenges and harness the full potential of AI in transforming PE diagnosis and management.

## 8 Conclusion

Integrating AI into CTPA for pulmonary embolism detection represents a promising frontier in medical imaging. AI algorithms demonstrate impressive potential in enhancing accuracy, efficiency, and accessibility in diagnosing pulmonary embolism. However, while AI offers significant advantages, its implementation must be free of limitations such as data quality, interpretability, and ethical considerations. Collaborative efforts among clinicians, radiologists, data scientists, and regulatory bodies are imperative to ensure AI technologies’ responsible and effective deployment in CTPA-based pulmonary embolism detection. By addressing these challenges, AI has the potential to revolutionize the diagnosis of pulmonary embolism, thereby enhancing patient outcomes and advancing the delivery of healthcare.
